# Supplementary material for: Anxiety symptoms and coping strategies used by older adults during COVID-19: A national e-study of linkages among and between them
Source: PLOS Ment Health. 2025 Apr 29;2(4):e0000304. doi: 10.1371/journal.pmen.0000304 (PMC12798459; doi:10.1371/journal.pmen.0000304)
Supplement: Attachment — Submitted filename: Response to Reviewers.docx [file pmen.0000304.s002.docx]

November 1, 2025

Charlene Sunkel

Editor-in-Chief, *PLOS ONE MENTAL HEALTH*

Founder and CEO, Global Mental Health Peer Network,

Paarl, Western Cape, ZA

Rochelle Burgess

Editor-in-Chief, *PLOS ONE MENTAL HEALTH*

Associate Professor, Institute for Global Health,

University College, London, GB

Ms. Sunkel and Dr. Burgess,

We thank you for this opportunity to revise and resubmit our research article entitled “Anxiety symptoms and coping strategies used by older adults during COVID-19: A national e-study of linkages among and between them” PMEN-D-24-00253. We are most thankful for the time that our reviewers have spent helping us improve our research article.

It is our pleasure to provide a point-by-point response to reviewer suggestions and questions.

**Reviewer #1:**

This study employed Network Analysis to create a visual representation of older Canadians’ symptoms of anxiety experiences and another of the linkages between their most pernicious symptoms and the strategies they used to cope with them. It provides a clear picture to show how older people managing or mitigating anxiety during the COVID-19 pandemic. Here are some suggestions for authors’ considerations:

1) Additional justification is required to substantiate the findings that depict the situation of older adults during the pandemic, given the absence of a 'control' group for comparison.

We thank you for your inquiry. Our study took place during a particularly frightening time in the pandemic - when social distancing just lifted and COVID-19 still lingered. After having been through lockdowns and mandated social distancing, older Canadians were being asked to transition into open social spaces while living most at risk for being hospitalized and dying from COVID-19. This was a very tall ask. When our study took place, researchers did not know what this transition would look like, let alone whether, when, and how it would unfold. Our asking older Canadians to complete an e-survey while making this transition was one other burden to bear. Being part of a control group would add to this burden. That is why we sought and recruited a nationally representative studied sample in proportion with Statistics Canada’s Population Census strata.

We also wish to point out that Network Analysis (NA) is a rather novel analytical technique absent in Canadian studies in the year our study took place and still now. Hence throughout our discussion, we point out how our findings support others’ findings from the first year of the pandemic and these are based on different statistical analytical techniques. Examples include our relating the troubling trio of anxiety symptoms to the prevalence of pernicious anxiety among older Canadians (page 12, para 1). Other instances are the observed positive associations between seeking credible information about COVID-19 and tuning in and breaking away from news (page 12, para 2), and the negative association between being kind to yourself and seeking credible information (page 13, para 2 and 3). We also point out novel associations, such as with breaking away from news and eating healthy, which warrants further research (page 14, para 1).

We present our findings without claims to causality; hence, our use of cautious language like ‘suggests’ (page 12, para 3) and ‘can be’ (page 15, para 3) and ‘could be’ (page 16, para 1). In our Conclusion section (page 15 para 3 and into page 16 para 1), we point out that network analysis blueprints are not prescriptive, rather they are a visual tool with which to compare one’s own anxiety and linked coping strategy. Our study is also a cross-sectional study, without pre- and post-test measurement concessions typical of studies involving a control group.

2) Given that COVID-19 is an unprecedented event, how the findings related to COVID-19 could inform scholars in the future and be applied to areas beyond the outbreak?

This adds further insights to our manuscript that readers can benefit from, as we have. In our Conclusions section, on page 17 (para 2), we therefore now state:

“How large-scale traumatic events unfold, and people’s reactions and remedial resources at-hand vary over time [85]. The COVID-19 pandemic has been described as sending unprecedented socioeconomic and health-related shockwaves through everyday people’s lives [1,2]. Its psychological repercussions are not fully realized [34,86]. In the first two years of the COVID-19 pandemic, older Canadians were living most at risk for falling gravely ill [8] and were living at arm’s length from loved ones [87]. Other older people stand to gain credible anticipatory guidance for managing pernicious anxiety when transitioning into open social spaces during a future pandemic. Researchers stand to gain lived insights with which to interpret their own findings, both now and in a future pandemic, and perhaps among other populations like older people acting to combat worrisome global climate change and to dampen their eco-anxieties [88].”

Accordingly, we have added four new references [85-89] to our reference list as well.

3) Please explain the distinctions between Network Analysis and traditional methods like logistic regression beyond just enhancing data visualization.

Readers will appreciate receiving this information as a methodological reflection. Hence, in our Implications on page 15, para 2, we now state: “Network analysis offers advantages that traditional multivariate analytical methods like logistic regression do not. Network analysis examines variable associations simultaneous with, rather than in isolation of one another [78]. People are not likely to experience one symptom of anxiety at a time, but rather a host of symptoms [79]. Anxiety symptoms most closely affiliated with one another can also be identified beside each other for subsequent testing in networks with other variables [80]. We now understand that older Canadians in this study were responding to a troubling trio of anxiety symptoms using multiple remedial strategies, seemingly with varying success. Network analysis embraces and offers nuanced understandings of the complexities of mental health recovery work [78,80]. Good COVID-19 research helps everyday people, practitioners, and program planners take stock of what others have learned at key points in time [32].”

Accordingly, we now also include in our reference list:

78. Briganti G, Scutari M, Epskamp S, Borsboom D, Hoekstra RHA, Golino HF, Christensen AP, Morvan Y, Ebrahimi OV, Costantini G, Heeren A, van Bork R, de Ron J, Bringmann LF, Marsman M, Blanken T, Gilbert A, Rhine Henry T, Fried EI, McNally RJ. Network analysis: An overview for mental health research. Int J Methods Psychiatr Res. 2024 Sep; 33(3): e2034. <https://doi.org/10.1002/mpr.2034>

79. Balsamo M, Cataldi F, Carlucci L, Fairfield B. Assessment of anxiety in older adults: a review of self-report measures. Clinical Interventions in Aging. 2018 Apr 6;13: 573-93. <https://doi.org/10.2147/CIA.S114100>

80. Borsboom D, Deserno MK, Rhemtulla M, Epskamp S, Fried EI, McNally RJ, Robinaugh DJ, Perugini M, Dalege J, Costantini G, Isvoranu A-M, Wysocki AC, van Borkulo CD, Waldorp LJ. Network analysis of multivariate data in psychological science. Nat Rev Methods Primers. 2022 Feb 21; 1:58. <https://doi.org/10.1038/s43586-021-00055-w>

4) The conclusion section is too sketchy, limitations about the study should be added.

We originally included study limitations in a paragraph just prior to the ‘Conclusions’ section on page 16, which read as:

“This study has limitations, however, including our reliance on data collected at one point in time. We found associations between and among symptoms and coping behaviours. We cannot say with any degree of certainty that a troubling trio of anxiety symptoms evoke any one strategic action, nor a chain reaction in others.

While our observed network associations provide valuable insights, it would be important to investigate their directionality. For instance, does increasing resilience decrease feelings of tension or lack of control, or do these feelings prompt individuals to become more resilient? Thus, it is also crucial to consider external factors or other internal processes that might influence these associations. This includes life events, other coping strategies not in the network, or sociodemographic factors like personal income [72] and personality [27]. Research also tells us that older people have a capacity to better regulate negative emotions [27, 83], even through the simplest domestic tasks [28] and by taking disrupted routines in stride [84]. Acknowledging and expressing negative emotions helped older Canadians persevere early in the pandemic [71].”

We have added these limitations to the Conclusions section on page 16 into para 3 and para 4 and have renumbered the references into both paragraphs to reflect the three new works addressing point #3. Doing so lends further credence to our Conclusions section.

We sincerely thank Ms. Sunkel and Dr. Burgess for granting us a revise-resubmit opportunity. All authors affirm that our newly revised article is original and no parts of it have been published elsewhere. Our desired venue for publication is *PLOS ONE MENTAL HEALTH*. All authors have approved and declare no known conflicts of interest with this article. I have changed my particulars to reflect my current affiliation and email address.

Sincerely,

Gail Low, RN, PhD, Associate Professor, Chair in International Health, Faculty of Nursing

MacEwan University, Edmonton; [lowg4@macewan.ca](mailto:lowg4@macewan.ca)
