# Supplementary material for: Anxiety symptoms and coping strategies used by older adults during COVID-19: A national e-study of linkages among and between them
Source: PLOS Ment Health. 2025 Apr 29;2(4):e0000304. doi: 10.1371/journal.pmen.0000304 (PMC12798459; doi:10.1371/journal.pmen.0000304)
Supplement: Attachment — Submitted filename: FEB21Response to Reviewers.docx [file pmen.0000304.s003.docx]

February 14, 2025

Charlene Sunkel

Editor-in-Chief, *PLOS ONE MENTAL HEALTH*

Founder and CEO, Global Mental Health Peer Network,

Paarl, Western Cape, ZA

Rochelle Burgess

Editor-in-Chief, *PLOS ONE MENTAL HEALTH*

Associate Professor, Institute for Global Health,

University College, London, GB

Ms. Sunkel and Dr. Burgess,

We thank you for you this opportunity to revise our manuscript entitled “Anxiety symptoms and coping strategies used by older adults during COVID-19: A national e-study of linkages among and between them” PMEN-D-24-00253R1. Paying heed to insightful reviewers’ comments and recommendations has certainly enhanced the quality of our work and help us put our best foot forward to entice PLOS ONE Mental Health readers. We provide detailed responses below.

Reviewer #1: Thank you. I do not have further comments.

We thank you for taking the time to review our work. We cannot spread the word about the importance of mental health scholarship without your kind and generous support.

Reviewer #2: The conducted study is of great interest and, despite the time that has passed since the pandemic, remains relevant. Complex behavioral and emotional regular mechanisms involved in the population's reactions to the unprecedented conditions of the pandemic remain incompletely understood. The chosen method of sample stratification and the authors' focus on the population of elderly respondents as a vulnerable group are of great value. Also valuable is the authors' practice-oriented approach to the problem under study and the desire to offer a visual tool for improving anti-stress mechanisms in the target audience. The ethical aspect of the study, including personalized questionnaires with password access, as well as financial support for respondents, is commendable. At the same time, a number of aspects of the work require clarification or supplementation.

**1.** The authors define the target audience for the application of the research results as elderly Canadians, but the article does not provide sociodemographic information about the sample for which the findings are applicable. What is the age dispersion, gender composition, economic level of the surveyed respondents, their employment and living conditions in the family? Without these data, the applicability of the study results is significantly limited.

In our original submission, in ‘Results’, p. 9, we alerted potential readers that “Our 1,327 e-survey responders are described in detail elsewhere [S2 File].” S2 is a supplementary *Psychogeriatrics* publication describing our studied sample in detail demographically along with their self-identified anxiety symptom scores and coping strategies (<https://doi.org/10.1111/psyg.13103>). We did this to avoid duplicate reporting of findings, and for transparency around other published work about our studied sample.

We do, however, see that readers would appreciate a ‘who’s who’ in which to situate our Network Analysis. We now alert potential readers to a ‘Sample Characteristics’ Table 1, a studied sample very nearly mirroring the general population of older Canadians in terms of age, sex, education, and gender. The anxiety-related significance of these factors is published in our S2 File.

Accordingly, on page 8 in para 1 of the “Results” section, we now state the following: “A total of 1,327 “YES” responders took our e-survey. They were strikingly similar in terms of age, sex, and education to the general population of older Canadians’ (Table 1). The coping strategies they identified with are shown in Table 2.”

**Table 1. Characteristics of studied sample (N = 1,327).**

| ^a^Frequency proportions do not sum to 100% due to missing values.  ^b^Frequency proportions falling below Statistics Canada Census Proportion. |  |
| --- | --- |
|  |  |

**Table 2. Self-identified Coping Stress and Anxiety** **Checklist strategies (N = 1,327).**

| ^a^Frequency proportions do not sum to 100% due to missing values.  ^b^Frequency proportions falling below Statistics Canada Census Proportion.” |  |
| --- | --- |
|  |  |

**2**. In terms of the applicability of the study results, the fact that the authors do not provide data on anxiety symptoms and coping strategies, but on their interrelationships, also requires a more in-depth analysis in the article. The manuscript does not contain information on the prevalence or intensity of symptoms, but only on their interrelationships. There is also no data on the frequency or intensity of coping strategies. Thus, not only should the title of the article be reformulated with an emphasis on the "network model of anxiety symptoms and coping strategies", but the discussion can also be built on possible explanations for the associations obtained, rather than an analysis of the vulnerability of the target audience.

We thank you for bringing this to our attention. Our new Table 1 and Table 2 (on page 8 and 9, ‘Results’ section) describes older Canadians’ perceived intensity of anxiety symptoms and coping strategies they self-identified with. This provides us with a much-needed context in which to further situate our ‘Discussion’ of findings. We were fortunate enough to learn from people having well-rounded perspectives about anxiety. Strikingly similar proportions of people were not at all and severely anxious. This addition reinforces the importance of attenuating our language in our discussion of ‘Positively connected coping strategies’ (page 12-13) and ‘Negatively connected coping strategies’ (page 13-15). We very much need to avoid an over-arching ‘old’ means vulnerable narrative in our discussion.

For example, on page 12, para 5, we originally stated: “Our associations suggest that credible sources might curb people seeking out news elsewhere. This is important because anxiety comes from realistic dangers and information from assumptive or misinformed sources [57, 58].

We now more aptly point out that: “Our associations suggest that credible sources might curb people seeking out news elsewhere. This kind of proactive constructive approach is important given that assumptive or misinformed sources have a penchant for fuelling, not quiescing, anxiety [59, 60].” Please note that the bracketed numbers differ here because we cited two new scholarly works on page 7 of our manuscript.

On page 13, para 2, we originally stated: “While some research [33] indicates some older people made strategic use of their resources to lessen loneliness and isolation and some [25] describe ‘pivoting’ or substituting pre-COVID routines with novel activities to stay mentally well, our responders did so through strategic pairing, with in tandem ‘*eating healthy’ and ‘staying active’* perhaps being another means by which respondents further minimise loved ones’ stress.

We now add: “While some research [33] indicates some older people made strategic use of their resources to lessen loneliness and isolation and some [25] describe ‘pivoting’ or substituting pre-COVID routines with novel activities to stay mentally well, our responders did so through strategic pairing, with in tandem ‘*eating healthy’ and ‘staying active’* perhaps being another means by which respondents further minimise loved ones’ stress. In this view, older Canadians are of generous spirit.”

On page 13, para 4, we originally stated, “Sharing negative news appears to trigger anxiety [65]. In the context of this study, perhaps being kind to yourself means shielding yourself from distressing facts, particularly while moving into open spaces at a heightened risk for physical health harms.”

We now more aptly point out that: “Sharing negative news appears to trigger anxiety [65]. In the context of this study, being kind to yourself through shielding yourself from distressing facts represents another constructive approach to moving back into open social spaces after two years of pandemic restrictions.”

In relation to the ‘*tuning in and breaking away from news’ and ‘eating healthy’ working in opposition to one another,* on page 14, we also now state: “As with ‘being kind to myself’, these are cross-sectional associations. Future qualitative research is warranted to better understand these relationships. We wondered whether older people were more attuned to the consumption of pandemic versus pantry staples. Prevailing news narratives centred around their vulnerability [55]. On the other hand, eating healthy might be a distraction, dulling one’s appetite for news policing or for any news at all.”

All such amendments afford vulnerability lesser social notoriety. When our study took place, Lagacé and colleagues (2022) warned us not to paint older people at large with the same broad vulnerability brush, and rightly so.

Relatedly, on page 16, para 3, we also now tell potential readers: “Our speaking of a troubling trio also shows the human side of anxiety management, with its trials and tribulations, and relatable imperfections. Everyday people need to be able to relate to research findings to talk about and learn from them [83]. We focus on symptoms and symptom-strategy relationships in our Network Analysis. This bird’s eye view avoids a certain age or sex group from, for example, being singled out as anxiety laden. Our findings are meant to help older people have less self-conscious (not conspicuous) recovery work conversations with clinicians. Ideally, taking stock of peers’ means of mitigating anxiety conjures taking stock of one’s own. Being a member of a population linked to COVID-19 [56] and mental health [84] are a social taboo. Why shouldn’t ‘old means vulnerable’ [85] have such notoriety?”

We added Lagacé to the reference list: [85] Lagacé M, O’Sullivan T, Dangoisse P, Mac A, Oostander S, University of Ottawa, Doucet A, Université du Québec à Montréal (2022). A Case Study on Ageism during the COVID-19 Pandemic; 2022 [cited 2025 January 28]. Available from. <https://cnpea.ca/images/ageism-covid19-en.pdf>

For these reasons, we also kindly request keeping the original title of our paper.

**3A)** The explanations of the associations in the manuscript require more correct formulations. As the authors correctly clarify in the limitations section, causal relationships cannot be established in a cross-sectional study. In this case, the interpretation of the obtained data is performed by the authors with an implicit assumption that coping strategies determine the development of anxiety symptoms: “Our respondents’ remember resilience seemingly strongly shielded them from feeling restless, tense, and as if they had no control over their lives”; “older Canadians in this study were responding to a troubling trio of anxiety symptoms using multiple remedial strategies, seemingly with varying success”.

You raise important points here. The notion that strategies can provoke symptoms and that symptoms can also provoke certain coping strategies fits with leading Network Analysis experts [50, 80] cautionary tales that observed relationships in a cross-sectional Network Analyses cannot tell us which variable is the true instigator. We had mentioned this on page 15, para 2 (see line 1, ‘vice-versa’), but with clipped vigor, given your concerns here.

For these reasons, we now state the following:

(1) On page 15, para 2: “Resilience was also important because a ‘*lack of control over my life*’ seemed to either deter ‘*staying active*’, or vice versa. Resilient older people are likely to be flexible about how often they stay active and what they gain from it. Simply getting outside has also brought older people closer to nature [76] and has given them some kind of social life [33] and helped them feel less isolated in general [71]. This kind of reframing has led others to find contentment with small social circles [19, 61] and in befriending technology [24]. Again, both scenarios are plausible. Observed relationships in a cross-sectional Network Analyses cannot tell us which variable is the true instigator [50,80].”
(2) On page 15, para 3: “The same can be said of respondents’ remembering resilience strongly shielding them from feeling restless, tense, and as if they had no control over their lives. This troubling trio may trigger memories of overcoming other hardships.”
(3) On page 16, para 1 we further reiterate: " Older Canadians in this study engaged in multiple remedial strategies to manage a troubling trio of anxiety symptoms. The presence and intensity of these symptoms may also influence the selection and perceived effectiveness of these coping strategies, suggesting reciprocal rather than strictly unidirectional relationships."
(4) On page 16, para 2, we now point out: “Taken together, the findings of this study tell us that mental health recovery work requires a wide variety of coping strategies, with some working in tandem and some in opposition. Symptoms themselves could widen this variety. Recovery work is hard work, particularly in the presence of pernicious anxiety symptoms.”

**3B)** Perhaps, it would be more effective to build a discussion through an analysis of effective and ineffective coping strategies, where ineffective strategies CAN provoke symptoms of (UNHEALTHY and HEALTHY) anxiety. Likewise, symptoms of (healthy) anxiety associated with the objectively existing threat of a pandemic CAN provoke the choice of ineffective coping strategies instead of effective ones, which reflects the maladaptive potential of the pandemic.

We thank you for sharing these sentiments. In keeping with our detailed response to point #2, our discussion is built around possible explanations for the associations obtained, without vulnerability (the potential for maladaptivity) as an over-arching narrative. We do not yet have strong consensus in the literature for these two categories. Anxiety is typically considered along a continuum, with adaptive and maladaptive aspects depending on context. Balsamo et al. [79] implore researchers to view anxiety experiences in later life as unique as older people’s life circumstances. Craske and colleagues (2009) remind us that anxiety should be understood as a continuum, ranging from a functional response to a pathological one. In keeping with our aim, capturing ranges of symptoms would be instructive for clinicians and for older people themselves. The older Canadians in our national snapshot study self-identified with a unique range of anxiety symptoms with a view to helping their peers learn from them. For these reasons, we have not dichotomized their anxiety scores as HEALTHY and UNHEALTHY to examine relationships.

Please see: Craske MG, Rauch SL, Ursano R, Prenoveau, J, Pine DS, Zinbarg RE. What is an anxiety disorder? Depression & Anxiety. 2009 Summer;26(12):1066–85. <https://doi.org/10.1002/da.20633>

**4.** Smaller, but also important for improving the manuscript, comments can be taken into account by the authors:

**4A)** the figures with the results of network analysis do not contain numerical values, which does not allow the reader to independently interpret the data. The strongest connections should be marked not only by the thickness of the lines, but also by numerical values. Otherwise, it is not obvious why the authors avoid discussing such anxiety symptoms as 5: Not control my worry and 10: Apprehensive. Unlike more traditional methods, such as factor and regression analysis, the proposed network analysis method does not have consensus "cutoff lines" and reliability levels, or they are not clearly enough presented in the article. Therefore, all connections (or at least half of the most significant of them) require explanation. Symptoms 5 and 10, according to the data in Figure 1, look more significant than symptom 9: No control over my life. According to Figure 3, their differences do not look significant enough. Similarly, according to Figures 3 and 4 for coping 4: Feeling anxiety is normal and coping 10: Challenging worries and anxious thoughts, it is necessary to provide an explanation why they are not included in the discussion (especially important in the context of comment #3 above).

We appreciate the reviewer’s comments and the opportunity to clarify the role of centrality indices in our analysis. In Network Analysis, symptoms with high centrality may exert a stronger influence on other symptoms within the network due to the strength and number of their connections (Borsboom & Cramer, 2013). Identifying these highly central symptoms is particularly relevant in clinical contexts, as they may serve as key targets for intervention (Borsboom & Cramer, 2013).

However, not all nodes in a network contribute equally to its overall structure or to its dynamic properties when processes operate within it (Costantini et al., 2015). Centrality indices serve as quantitative measures of a node's importance, capturing its role within the network based on the pattern and strength of its connections. Consistent with this rationale, empirical studies have widely used centrality indices to determine the importance of symptoms within a network structure (Borsboom et al., 2021). The vast majority of network analysis studies do not display numerical values for individual connections in graphical representations but instead prioritize the interpretation of centrality indices to highlight the most influential symptoms (e.g., Samra et al., 2023; Borsboom et al., 2021; França et al., 2020; Robinaugh et al., 2020) and network stability analysis (to assess the robustness of results) (Borsboom et al., 2021).

In these respects, please see:

Borsboom D, Cramer, AO. Network analysis: an integrative approach to the structure of psychopathology.  Ann Rev Clin Psychol. 2013 Mar; *9:*91–121. [https://doi.org/10.1146/annurev-clinpsy-050212-185608](https://doi.org/10.1146/annurev-clinpsy-050212-185608 )

Costantini, G., Epskamp, S., Borsboom, D., Perugini, M., Mottus, R., Waldorp, L. J., & Cramer, A. O. J. (2015).

State of the aRt personality research: A tutorial on network analysis of personality data. J Res Personality. 2015 Feb;54:13–29. <https://doi.org/10.1016/j.jrp.2014.07.003>

Borsboom, D., Deserno, M. K., Rhemtulla, M., Epskamp, S., Fried, E. I., McNally, R. J., Robinaugh, D. J., Perugini, M., Dalege, J., Constantini, G., Isvoranu, A. M., Wichers, M., & Cramer, A. O. J. (2021). Network analysis of multivariate data in psychological science. Nat Rev Methods Primers. 2021;1:58. <https://doi.org/10.1038/s43586-021-00055-w>

França AB, Gordon AL, Samra R, Duarte, ESR, & Jacinto AF. Symptoms of mood disorders in family carers of older people with dementia who experience caregiver burden: A network approach. Age Ageing, 2020 Sept;49(4):628–633. <https://doi.org/10.1093/ageing/afaa031>

Robinaugh DJ, Hoekstra RHA, Toner ER, Borsboom D. The network approach to psychopathology: a review of the literature 2008-2018 and an agenda for future research. Psychol Med 2020 Dec;50(3):353–66. <https://doi.org/10.1017/S0033291719003404>

Samra R, França, AB, Lucassen MFG, Waterhouse P. A network approach to understanding distance learners’ experience of stress and mental distress whilst studying. Int J Educ Tech Higher Educ. 2023 May;20(1), Article 27. <https://doi.org/10.1186/s41239-023-00397-3>

Following this established approach, we focused our discussion on symptoms with the highest centrality values rather than on individual edge weights. However, based on the reviewer’s valuable suggestion, we have now expanded our “Network Description and Node Characteristics” section to clarify why certain symptoms were prioritized. More specifically, starting on page 7, para 3 up to page 8, para 1, we emphasize:

“We evaluated the relative importance of nodes representing GAS-10 anxiety symptoms using Strength Centrality, which is the sum of all the weights connected to a symptom in absolute value. This index tells how well connected a particular anxiety symptom is to all other nodes or GAS-10 symptoms in the network, meaning they play a key role in maintaining and potentially exacerbating anxiety symptomatology [45]. In line with previous studies, we focused our interpretation on symptoms with the highest centrality values rather than on individual edge strengths, as this approach is commonly used in empirical network analysis research [46]. Strength Closeness and Betweenness were not reported because neither have generally shown stability and so they can be problematic measures since they treat associations as, distances [47,48].”

Our reference list aptly reflects this page 7, para 2 amendment:

45. Borsboom, D., & Cramer, A. O. (2013). Network analysis: an integrative approach to the structure of psychopathology. Annu Rev Clin Psychol. 2013 Mar; 9*:*91–121. [https://doi.org/10.1146/annurev-clinpsy-050212-185608](https://doi.org/10.1146/annurev-clinpsy-050212-185608 )

46. Borsboom, D., Deserno, M. K., Rhemtulla, M., *et al*. Network analysis of multivariate data in psychological science. Nat Rev Methods Primers. 2021;1:58. [https://doi.org/10.1038/s43586-021-00055-w](https://doi.org/10.1038/s43586-021-00055-w )

 47. Epskamp S, Borsboom D, Fried EI. Estimating psychological networks and their accuracy: A tutorial paper. Behav Res Methods. 2017 Mar 24;50:195-212. <https://doi.org/10.3758/s13428-017-0862-1>

48. Hallquist MN, Wright AG, Molenaar PC. Problems with centrality measures in psychopathology symptom networks: Why network psychometrics cannot escape psychometric theory. Multivariate Behav Res. 2019 Aug 12;56(2):199-223. <https://doi.org/10.1080/00273171.2019.1640103>

**4B)** The discrepancy in the number of respondents surveyed given in the abstract (1,327) and in the caption to Figure 1 (n = 1,224) requires an explanation. Perhaps there were socio-demographic characteristics of the respondents not included in the network analysis for constructing the figures?

We thank you for your inquiry. The 1,327 older Canadians responding to our e-survey answered as many or as few questions as they wanted to (see page 6, para 1). Hence, our lesser (n =1,224) Network Analysis sample size reported alongside Figure 1. Our responders were transitioning into larger open spaces, with COVID-19 still lingering and being most at risk for hospitalizations and dying. Forced responses would be unethical. Missingness is alluded to on page 8 “Results” (i.e., being strikingly similar), and in Table 1 and Table 2. We expect that other researchers will find this instructive for future pandemic research.

Sociodemographic characteristics were not included in our Network Analyses, for good reasons.

As we now state on page 16, para 3, in our “Implications” section: “Our speaking of a troubling trio also shows the human side of anxiety management, with its trials and tribulations, and relatable imperfections. Everyday people need to be able to relate to research findings to talk about and learn from them [83]. We focus on symptoms and symptom-strategy relationships in our Network Analysis. This bird’s eye view avoids a certain age or sex group from, for example, being singled out as anxiety laden. Our findings are meant to help older people have less self-conscious (not conspicuous) recovery work conversations with clinicians. Ideally, taking stock of peers’ means of mitigating anxiety conjures taking stock of one’s own. Being a member of a population linked to COVID-19 [56] and mental health [84] are a social taboo. Why shouldn’t ‘old means vulnerable’ [85] have such notoriety?”

**4C)** An addition is required in the limitations section about the applicability of the results only to elderly residents of Canada, or the authors' arguments as to why the patterns found can be considered universal, and therefore in some part applicable (under what conditions) to wider target audiences

We thank you for raising this point. We cannot generalize to older people residing in different countries.

We therefore now state on page 17, in our “Conclusions” section: “This study has limitations, however, including our reliance on data collected only in Canada, and at one point in time.”

On page 18, we also now state: “Contemporaries’ anticipatory guidance for managing pernicious anxiety when transitioning into open social spaces during a future pandemic should resonate with other older Canadians and offer points of comparison for age-peers outside of Canada [88].”

In closing, we thank our *PLOS ONE Mental Health* reviewers for their time and attention. We trust that our revised manuscript addresses reviewers’ thoughtful recommendations. Please do not hesitate to contact me should you have any questions about PMEN-D-24-00253R1. It would be an honor to publish this work in your seminal journal.

We included updates ethics approval renewal letters on the *PLOS ONE Mental Health* submission platform, and our approval numbers in our manuscript (p. 19). We have a new author (A. Naz) who is a PhD candidate and trainee in mental health promotion, and for whom we have added credentials (p. 1) and contributions (p. 19). We support up-and-coming researchers. Mental health matters to us all (<https://www.who.int/publications/i/item/9789240049338>).

Sincerely,

Gail Low, RN, PhD

Associate Professor, Faculty of Nursing

MacEwan University, Edmonton; [lowg4@macEwan.ca](mailto:lowg4@macEwan.ca)
